# Cardiovascular prognosis: a new role for ceramides and other cardiometabolites

**DOI:** 10.1002/ehf2.13025

**Published:** 2020-10-11

**Authors:** Jasmine M. F. Wu, P. Christian Schulze

**Affiliations:** ^1^ Division of Cardiology, Department of Internal Medicine I University Hospital Jena Jena Germany

One of the hallmarks of cardiovascular diseases is metabolic perturbation, and one of the circulating metabolites that have drawn much attention in recent years is the subclass of ceramides. Ceramides are a class of sphingolipids composed of sphingosine and a fatty acid. Ceramide levels are elevated in the heart failure patients, suggesting a connection between ceramide metabolism and progression of cardiovascular diseases.^1^ In this issue of the journal, Targher *et al*. conducted a study analysing 400 chronic heart failure (HF) patients to examine the link between ceramide levels and cardiovascular mortality.^2^ The study is based on a comparative analysis, which was conducted between survivors and non‐survivors with pre‐existing HF over a median follow‐up period of 3.9 years.

Increased cellular ceramides have been linked to enhanced apoptosis, insulin resistance, and oxidative stress.^3^, ^4^, ^5^, ^6^, ^7^, ^8^ Ceramides are synthesized through three major pathways: first, *de novo* condensation of palmitoyl CoA with serine catalyzed by serine palmitoyltransferase (SPT),^9^ second, by sphingomyelinase‐dependent hydrolysis of sphingomyelin,^10^ and third, from sphingosine through ceramide synthases (*Figure* *1*). *De novo* synthesis contributes 25–30% of total ceramides and is activated in inflammation and hypoxia.^11^, ^12^, ^13^ Two subunits of SPT (SPT1 and SPT2) form a heterodimer and are both necessary for enzyme function. Specificity of these pathways for ceramide subspecies is unclear; formation of long‐chain ceramides (C22–C26) has been linked to ceramide synthase 2 (CerS2),^14^, ^15^ and long‐chain ceramides and very‐long‐chain ceramides are believed to have the greatest impact on cardiac dysfunction.^1^, ^16^ Our previous studies demonstrated that ceramide accumulation causes cardiac remodelling and ultimately failure.^1^, ^16^ Saturated fat increases ceramides,^17^, ^18^ and genetic and pharmacological inhibition of ceramide synthesis ameliorates insulin resistance,^5^ a hallmark of HF. In an earlier study, Yu *et al*. showed that total ceramide levels had a positive correlation with cardiovascular morbidity and increased mortality rates.^19^ By comparison of ceramide levels between the two chronic HF patient groups, Targher *et al*. revealed that only plasma Cer(d18:1/16:0) and Cer(d18:1/24:1) levels increased in the patients who died of cardiovascular diseases. Furthermore, an unadjusted comparison showed that higher ratios of each ceramide with Cer(d18:1/24:0) were significantly associated with increased mortality. Adjustment for additional cardiovascular risk factors, however, weakened the association, in particular after adjustment for levels of plasma NT‐proBNP and PTX3.^2^

**Figure 1:**
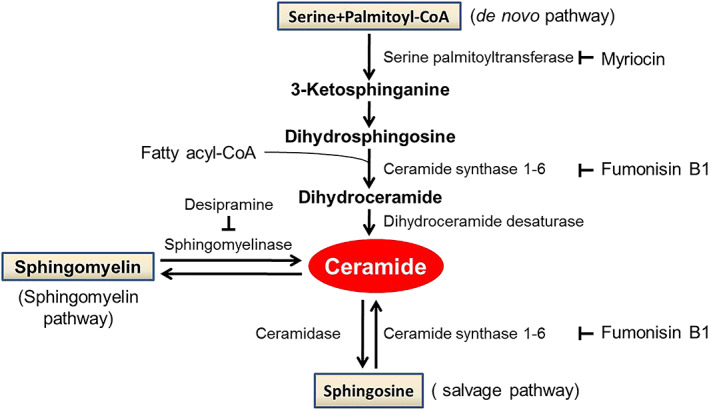
Pathways of ceramide synthesis and degradation. Ceramide *de novo* synthesis from serine and palmitate is catalyzed by serine palmitoyltransferase and contributes to around 30% of the total ceramide pool. This pathway is activated in hypoxia and inflammation and is inhibited by myriocin. The salvage pathway forms ceramides from sphingosine catalyzed by ceramide synthases (CerS1–CerS6) with tissue and ceramide species synthesis specificity. CerS1, CerS2, and CerS5 are all expressed in the myocardium, and CerS2 mediates synthesis of long‐chain ceramides C22–C24. This pathway can be inhibited by fumonisin. The sphingomyelin pathway is catalyzed by sphingomyelinase.

In the current study, the selected population of patients who later died from cardiovascular causes already had significantly higher levels of plasma PTX3 and NT‐proBNP at baseline. These two well‐documented prognostic biomarkers are associated with increased mortality and adverse cardiovascular events.^20^, ^21^, ^22^ Potentially, high plasma PTX3 and NT‐proBNP are dependent predictors of a positive association between ceramide levels and cardiovascular death. However, this requires further confirmation by a larger cohort of patients, preferentially between the patients with comparable levels of PTX3 and NT‐proBNP. Chronic HF is associated with elevated inflammatory state, especially in patients at advanced stages of the disease.^23^, ^24^ Considering that levels of PTX3 and NT‐proBNP as well as ceramides are regulated by specific immune responses,^25^, ^26^, ^27^ elevated levels of these three markers in patients with HF who later died of cardiovascular disease may arise from enhanced inflammation. Inclusion of additional adjustments for inflammatory parameters will allow to better evaluate the effect of ceramide levels on cardiovascular outcome in the future.

The fact that ceramide levels increase after heart injury renders its prognostic value to predict cardiovascular outcome. However, the results reported by Targher *et al*. implicate an impact of ceramide levels on cardiovascular events and should be evaluated in the context of other studies to avoid overestimation or underestimation of the outcomes. A better understanding of the pathways underlying the regulation of ceramide metabolism will certainly favour selection of suitable adjustment variables when evaluating effects of ceramides for clinical application.

Altogether, cardiometabolites are a class of biomarker molecules that have gained new attention not only as indicators of cardiovascular metabolism but also concerning their prognostic role and impact on cardiovascular outcomes. New powerful methods for the detection and quantification of these molecules will allow testing their specific role in routine clinical applications. Furthermore, definition of their role and impact will provide opportunities for novel pharmacological interventions in cardiovascular diseases and beyond.

## Conflict of interest

None declared.
